# Secondary Use of Health Data for Medical AI: A Cross-Regional Examination of Taiwan and the EU

**DOI:** 10.1007/s41649-024-00279-4

**Published:** 2024-04-06

**Authors:** Chih-hsing Ho

**Affiliations:** Institute of European and American Studies, Academia Sinica, Taipei, Taiwan

**Keywords:** Secondary use, Health data, Data governance, Medical AI, EHDS, Taiwan

## Abstract

This paper conducts a comparative analysis of data governance mechanisms concerning the secondary use of health data in Taiwan and the European Union (EU). Both regions have adopted distinctive approaches and regulations for utilizing health data beyond primary care, encompassing areas such as medical research and healthcare system enhancement. Through an examination of these models, this study seeks to elucidate the strategies, frameworks, and legal structures employed by Taiwan and the EU to strike a delicate balance between the imperative of data-driven healthcare innovation and the safeguarding of individual privacy rights. This paper examines and compares several key aspects of the secondary use of health data in Taiwan and the EU. These aspects include data governance frameworks, legal and regulatory frameworks, data access and sharing mechanisms, and privacy and security considerations. This comparative exploration offers invaluable insights into the evolving global landscape of health data governance. It provides a deeper understanding of the strategies implemented by these regions to harness the potential of health data while upholding the ethical and legal considerations surrounding its secondary use. The findings aim to inform best practices for responsible and effective health data utilization, particularly in the context of medical AI applications.

## Introduction

Data plays a pivotal role in the realm of medical AI, serving as the lifeblood that fuels its transformative potential (Rajpurkar et al. 2022). In the complex landscape of healthcare, the sheer volume and diversity of data generated daily in hospitals, clinics, and research institutions are invaluable resources. Medical AI systems rely on this data to learn and adapt, enabling them to make accurate diagnoses, predict patient outcomes, and optimize treatment plans. With access to electronic health records, medical imaging scans, genomic information, and even wearable device data, AI algorithms can detect subtle patterns, identify anomalies, and assist healthcare professionals in making informed decisions (Shaheen 2021). Moreover, as AI algorithms continually refine their understanding of medical data, they contribute to the advancement of personalized medicine, uncovering novel insights and tailoring treatments to individual patient needs (Johnson et al. 2021).

Nevertheless, the availability of existing medical data for AI training is a complex and often challenging endeavor. Two major obstacles that loom large are consent and the limitations of de-identification. Obtaining informed consent from patients for their data to be used in AI research is not only an ethical imperative but also a legal requirement in many jurisdictions. The process of obtaining such consent can be intricate, time-consuming, and may not always yield the desired outcomes, as patients may opt out or express reservations about data sharing (Meszaros and Ho 2019). Additionally, while de-identification techniques are employed to protect patient privacy, they are not foolproof, and there remains a risk of re-identification when dealing with rich, multifaceted medical datasets (Phillips and Knoppers 2016). Striking a balance between leveraging existing data for AI advancements and safeguarding patient rights and privacy is an ongoing challenge that healthcare institutions, researchers, and policymakers must grapple with to ensure that medical AI can reach its full potential while upholding ethical standards and legal obligations (Kerasidou and Kerasidou 2023).

This paper will delve into a comparative analysis of data governance mechanisms pertaining to the secondary use of health data in Taiwan and the EU. Both regions have distinct approaches and regulations concerning the utilization of health data for purposes beyond primary care, such as medical research, innovation, and healthcare system improvement. By examining these two approaches, this study aims to shed light on the strategies, data governance frameworks, and legal frameworks adopted by Taiwan and the EU to strike a balance between the imperative of data-driven healthcare innovation and the protection of individual privacy rights. This comparative exploration will offer valuable insights into the evolving landscape of health data governance on a global scale and provide a deeper understanding of the strategies employed by these regions to harness the potential of health data while safeguarding the ethical and legal considerations surrounding its secondary use.

## Constitutional Court Ruling on the Secondary Use of National Health Insurance Data in Taiwan

On August 12, 2022, the Taiwan Constitutional Court (referred to as the “TCC”) issued a significant judgement, identified as Judgment No. 13 (111-Hsien-Pan-13), which had far-reaching implications for the secondary use of national health insurance data within the country (TCC Judgment 111-Hsien-Pan-13 2022). This ruling concerned the National Health Insurance Administration’s (NHIA) practice of converting data from the national health insurance system into administrative data for secondary research purposes through de-identification, without obtaining individual consent or offering an opt-out option. In this judgement, the TCC upheld the constitutionality of Subparagraph 4, proviso of Paragraph 1, Article 6 of the Personal Data Protection Act (referred to as the “PDPA”). Simultaneously, it declared Articles 79 and 80 of the National Health Insurance Act (referred to as the “NHI Act”) unconstitutional (TCC Judgment 111-Hsien-Pan-13 2022). The TCC conducted a thorough examination, applying strict scrutiny. It determined that the contested PDPA provisions, which placed restrictions on data subjects’ rights to data protection for the purpose of healthcare, public health, or crime prevention with de-identified data, were found to be proportionate. However, the TCC emphasized the vital need for sufficient legal institutions, such as effective supervisory instruments for storing, processing, transmitting, and providing personal data (TCC Judgment 111-Hsien-Pan-13 2022). The absence of such mechanisms in the existing regime was identified as unconstitutional. Furthermore, the TCC highlighted the lack of regulations enabling data subjects to exercise their right to opt-out, considering this absence a violation of the Constitution. Consequently, the court has mandated that the responsible authority must establish the necessary legal mechanisms to uphold the constitutional right to personal data protection within a grace period of three years from the date of this judgement’s announcement (TCC Judgment 111-Hsien-Pan-13 2022).

In 1995, Taiwan introduced mandatory enrollment in the National Health Insurance (NHI) scheme for all citizens and working residents (Wu et al. 2010). To streamline reimbursement and financial tracking, healthcare institutions are obligated to provide data to the NHIA, resulting in the accumulation of significant personal data, including medical records, prescriptions, and images. The NHIA initially entrusted the National Health Research Institute in Taiwan to establish the National Health Insurance Research Database (NHI Database), accessible to external parties since 2000. The NHIA-NHRI collaboration ceased in 2016, with all original data returning to the NHIA. Subsequently, the NHIA established the Applied Health Research Data Integration Service and the Health and Welfare Data Science Center to oversee NHI data. Academic researchers can access NHI data upon request for research, subject to scrutiny (Chen 2019).

This case involves petitioners from Taiwanese NGOs who alleged the NHIA’s unauthorized sharing of personal NHI data for purposes beyond NHI enforcement violated data protection rights (Wu and Hetherington 2022). In 2012, the petitioners requested that the NHIA refrains from disclosing their NHI data to third parties, but the NHIA declined, citing sufficient data protection measures and a commitment to national welfare. After administrative appeals and legal proceedings, the petitioner’s case was dismissed by the Supreme Administrative Court in 2017. Subsequently, the petitioners initiated a constitutional review in the same year (TCC Judgment 111-Hsien-Pan-13 2022).

According to the TCC, Articles 79 and 80 of the NHI Act lack explicit regulations concerning critical aspects, such as the purpose, legal framework, scope, and procedures for preserving, processing, transmitting, and external provision of NHI data. They also fall short in addressing essential subjects such as independent supervisory instruments for organizational and procedural data protection matters (TCC Judgment 111-Hsien-Pan-13 2022). This regulatory gap infringes upon the constitutional protection of personal data guaranteed by Article 22 of the Constitution. Consequently, the competent authority is obliged to amend the relevant provisions in the NHI Act and other laws or establish specific legislation within the same three-year timeframe to explicitly address these matters (TCC Judgment 111-Hsien-Pan-13 2022). Regarding the secondary use of personal health insurance data beyond its original collection purpose, especially when shared with other government agencies or academic research institutes, the current legal framework lacks provisions allowing data subjects to opt out. This omission constitutes a violation of the constitutional protection. To rectify this, the competent authority must, within the three-year grace period, amend or establish relevant laws that explicitly define the subject, reasons, procedure, and effects of requesting (or exceptionally denying) opt-out. If the amendment or creation of these laws is overdue, individuals should be able to directly request opting out from the use of their NHI data (TCC Judgment 111-Hsien-Pan-13 2022).

Following the Constitutional Court’s ruling, the NHIA has taken proactive steps to initiate proposed legislation. This legislation aims to establish a specific law governing the secondary use of NHIA data, providing a legal foundation for its further processing outside of original purposes. Importantly, the proposed law includes provisions to allow individuals the option to opt out of certain data processing activities and outlines specific conditions that can restrict opt-out rights. Furthermore, as part of data governance, the NHIA is planning to institute a committee composed of individuals with diverse backgrounds and stakeholders. This committee’s primary responsibility will be to meticulously review data access requests, ensuring a balanced approach that incorporates a wide range of perspectives while upholding data privacy and security standards. This initiative reflects a comprehensive strategy to enhance data governance, fostering transparency, accountability, and responsible data utilization in line with legal and ethical standards.

However, while numerous efforts have been made to establish a robust legal framework through the constitutional court judgement, several studies indicate that relying solely on law is insufficient for effective data governance (Meszaros and Ho 2019; Horn and Kerasidou 2020). The key lies in building public trust through engagement. It is crucial to emphasize that encouraging people to willingly remain in the database, without opting out, necessitates a focus on fostering trust rather than solely relying on legal mechanisms. The failure of the care.data programme in the UK, for example, serves as a demonstration that a top-down approach to centralizing health data is ineffective without sufficient social trust (Carter et al. 2015). The NHS England faced criticism for inadequately communicating the programme’s purpose, resulting in over one million people opting out. The NHS care.data scheme was ultimately closed in 2016 following years of controversy. This underscores the importance of public engagement in data governance and highlights the necessity of earning people’s trust in such initiatives, especially through transparent communication about the government’s data use plan.

Furthermore, while proposing independent supervisory instruments in Taiwan, the emphasis remains on legal and institutional mechanisms. However, it is crucial to acknowledge that effective supervision, being a vital aspect of good governance, extends beyond mere legal requirements by incorporating ethical considerations. This perspective underscores a commitment to responsible and transparent practices, highlighting the significance of ethical conduct that surpasses the confines of legal mandates. As the NHIA's proposed law is currently under discussion, it remains challenging to accurately predict the potential opt-out rate in Taiwan once the law is enacted. The intricacies of data privacy and the evolving landscape of healthcare information necessitate careful consideration and public engagement to foster social trust. Maintaining a low opt-out rate is crucial for preserving the integrity and comprehensiveness of the database (Piel et al. 2018). To achieve this, it is imperative to involve the public in discussions, address concerns, and build a consensus that balances the benefits of healthcare data usage with robust data protection measures. Such inclusive efforts will be instrumental in promoting responsible data governance and ensuring that the healthcare data ecosystem continues to align with the interests of all stakeholders while maintaining stringent privacy and security standards.

## De-identification and Genomic Data Protection

While the court’s ruling has affirmed the constitutionality of processing pseudonymized data for public interest, it underscores that this alone is not a sufficient condition to justify the secondary use of health data without an opt-out mechanism. Particularly for extensive databases like the NHIA’s, obtaining opt-in consent from every individual is logistically challenging, making opt-out an essential alternative for individuals to exercise their autonomy over their data. This highlights the importance of a comprehensive data governance approach that extends beyond data de-identification. It emphasizes the need for supplementary mechanisms, such as opt-out options, to strike a balance between data utilization for societal benefit and respecting individuals’ privacy and autonomy rights.

In addition, in both the European Union’s General Data Protection Regulation (GDPR) and Taiwan’s PDPA, there is a distinct treatment of anonymized data, which is not subject to the stringent data protection rules. However, the challenge lies in achieving adequate anonymization, as technology continuously evolves, rendering the task complex and ever-changing (Carvalho et al. 2020). In Taiwan, further complicating matters is the lack of clear guidance on the definitions and distinctions between two critical terms—pseudonymization and anonymization. It is important to note that, under the GDPR, pseudonymized data is still considered personal data. In jurisdictions following the GDPR, obtaining consent remains a legal requirement for further data processing. In the USA, as outlined in the Health Insurance Portability and Accountability Act (HIPAA), the removal of certain identifiers is explicitly defined as a criterion for considering data as anonymized. These legal clarities serve to reduce uncertainty in daily practice. However, in Taiwan, it is often observed that data is highly pseudonymized, yet there remains the potential for re-identifying specific data subjects. In such instances, the data subject’s right to consent or opt-out is compromised as pseudonymized data is used, even though the data has not yet been adequately anonymized. This ambiguity has left data controllers and processors grappling with uncertainty, making it challenging to determine precisely when de-identified data can be considered non-personal and thus exempt from the data protection framework. Addressing these intricacies and providing precise guidance on data de-identification are vital in ensuring compliance with data protection regulations while fostering responsible data utilization practices.

The ongoing debate surrounding data anonymization becomes increasingly complex with the inclusion of genomic data into the NHIA’s database, particularly as the national health insurance system is set to start reimbursing next-generation sequencing (NGS) in Taiwan. The critical question arises: can genomic data ever be adequately anonymized? Genomic data, by its very nature, presents an intricate conundrum. It comprises a wealth of highly personal and sensitive information, encompassing an individual’s unique genetic makeup, susceptibility to diseases, and even potential hereditary conditions (Bonomi et al. 2020). This inherent specificity makes achieving adequate anonymization a formidable task. Unlike other forms of healthcare data, genomic information cannot be stripped of its individuality without compromising its utility for research and medical advancements. As the integration of genomic data into healthcare databases advances, it becomes imperative to address these complexities comprehensively (O’Doherty et al. 2021).

Striking the right balance between harnessing the potential of genomic data for research and healthcare advancements while safeguarding individual privacy and data protection rights remains a significant challenge and an essential aspect of evolving data governance practices. As Taiwan embraces the integration of genomic data, it faces an urgent need to grapple with these multifaceted challenges on a comprehensive scale. The central question of whether genomic data can ever adequately be anonymized underscores the complexity of genomic data management, demanding nuanced and adaptable solutions that reconcile the pursuit of scientific progress with the protection of individual privacy and autonomy. It calls for the establishment of robust frameworks that not only preserve data security but also empower individuals with clear insights into how their genomic information is used, by whom, and for what purposes. In addition to emphasizing de-identification, there have been extensive international discussions on the sharing of genomic data. For instance, the Global Alliance for Genomics and Health (GA4GH) has published guidelines to complement existing laws and regulations on privacy and personal data protection. These guidelines also address policies and codes of conduct for the ethical governance of research. The objectives include fostering responsible data sharing and oversight of research databases, as well as establishing a framework for enhanced international collaboration and good governance. Furthermore, the guidelines provide overarching principles to be respected in developing legally binding tools, such as data access agreements. This demonstrates that while de-identification is a crucial aspect of data protection, a comprehensive data governance system involves a broader set of principles and practices (Knoppers 2014). 

For a significant period, Taiwan has leaned heavily on data de-identification, often without explicit consent, to facilitate the secondary use of data for research purposes. This practice has traditionally found its justification in the broader concept of public interest, as leveraging healthcare data for research has undeniable societal benefits. However, as the landscape of healthcare evolves, particularly with the increasing involvement of public-private partnerships in endeavors like medical AI development, the limitations of relying solely on de-identified data are becoming evident. In the context of collaborative ventures involving commercial entities, there is a growing recognition that data subjects deserve greater transparency and control over the usage and access to their data (Meszaros and Ho 2021). While de-identification serves as a protective measure, it does not provide individuals with a comprehensive understanding of how their data is being utilized and by whom. As medical AI development increasingly relies on these collaborations, it is imperative to establish more transparent data governance mechanisms that not only safeguard privacy but also empower data subjects with detailed insights into the precise nature of data usage, the entities involved, and the purpose behind such utilization (Haibe-Kains et al. 2020).

Utilizing the GA4GH guidelines as an illustration, transparency entails developing clearly defined and easily accessible information concerning the purposes, processes, procedures, and governance frameworks for data sharing. This information should be presented in a manner that is comprehensible and accessible across both digital and non-digital formats. It involves furnishing explicit details on the purpose, collection, use, and exchange of genomic and health-related data. This encompasses aspects such as data transfer to third parties, international data transfer, terms of access, duration of data storage, identifiability of individuals and data, limitations to the anonymity or confidentiality of data, communication of results to individuals and/or groups, oversight of downstream uses of data, commercial involvement, proprietary claims, and procedures for withdrawing from data sharing (Rehm et al. 2021; Knoppers 2014). Balancing the imperative of healthcare innovation with the protection of individual privacy rights necessitates a shift toward more transparent and accountable data governance practices. This evolution is essential to ensure that public-private partnerships in medical AI development respect data subjects’ autonomy and foster trust, ultimately contributing to the responsible and ethical advancement of healthcare technology.

## Health Data Integration in the EU: The European Health Data Space

The European Health Data Space (EHDS) outlines a comprehensive framework within the European Union, aiming to facilitate the utilization of health data for various purposes such as research, innovation, public health, policy-making, regulatory activities, and personalized medicine. A key component of this initiative is the establishment of a decentralized EU infrastructure known as HealthData@EU, connecting health data access bodies across all Member States (European Commission 2022). In recent years, the rapid evolution of technology and the unprecedented challenges posed by the COVID-19 pandemic underscored the indispensable role of contemporary health data in shaping informed decisions on public health policies and crisis management. While the GDPR has been instrumental in safeguarding individuals’ rights concerning personal health data, there remain significant challenges in the exercise of these rights, particularly in the context of electronic health data (Marcus et al. 2022). It is within this backdrop that the EHDS emerges as a transformative initiative.

March 2022 marked a significant milestone with the proposal of the EHDS regulation. This ambitious initiative envisions a future where competent authorities can seamlessly connect disparate health data sets, rendering them more accessible and harmonized across the EU. At its core, the EHDS aims to not only enhance the primary use of health data for crucial aspects such as healthcare delivery but also to empower individuals, putting them firmly in control of their health data (Marcus et al. 2022). However, the EHDS’s true innovation lies in its approach to the secondary use of health data. It opens the doors to harnessing health data for purposes of innovation, scientific research, and evidence-based policymaking, thereby catalyzing advancements that promise to revolutionize healthcare (Marcus et al. 2022).

The EHDS data governance design includes key elements aimed at enhancing trustworthiness. Specifically, individuals seeking to reuse health data are obligated to obtain a permit from a health data access body, clearly defining the conditions and purposes for the data’s utilization. In addition, access to the data is restricted to closed, secure environments provided by these access bodies, adhering to strict cyber security standards. Users can extract only anonymous data from the secure processing environment, and in cases where access to personal electronic health data is necessary, it can only be obtained in pseudonymized form to protect individual identities. In order to increase transparency for data use, the EHDS requires health data access bodies to publish information about data access applications, while users must publicly disclose the results of their electronic health data uses. Furthermore, the EHDS promotes cross-border collaboration by mandating the participation of all Member States in the HealthData@EU infrastructure, fostering a unified approach to secondary data use within the EU and beyond. The infrastructure will undergo a pilot phase in a EU4Health project starting in 2022 (The European Health Data Space 2022).

The EHDS proposal introduces a comprehensive framework for the secondary use of health data. Article 33 outlines the minimum categories of data eligible for secondary use, including health impact data, human genetic data, health data registries, and clinical trials data. The proposal further delineates permitted and prohibited purposes in Articles 34 and 35, aiming to strike a balance between data utilization for beneficial ends and safeguarding individual rights. Permitted purposes encompass development and innovation activities, while prohibited purposes include detrimental use against individuals, advertising, data access to unpermitted third parties, and the development of harmful products. Within this framework, the proposal introduces distinct roles: “data users” and “data holders”. Data users are individuals or entities with lawful access to electronic health data for secondary use, while data holders encompass entities in healthcare, research, or EU institutions with the right to provide data under EU law. Notably, pharmaceutical companies can access data from entities like hospitals, even for commercial purposes, if it aligns with legitimate interests such as scientific research and innovation.

The process for secondary use entails data users submitting requests for access to health data, with the specifics varying based on the sought-after data (Articles 45, 47). These requests undergo evaluation by a health data access body, responsible for granting access permission and ensuring compatibility with the purposes outlined in Article 34(1). Given the sensitivity of electronic health data, measures are in place to mitigate privacy risks. Anonymized health data is preferred whenever possible. However, if personal data is essential and justified, it should be provided in pseudonymized format, with the encryption key held exclusively by a health data access body (Recital 49). This safeguards the privacy of individuals while facilitating the valuable secondary use of health data for critical purposes.

The EHDS, in conjunction with the GDPR, empowers individuals with specific rights concerning their health data. This includes the immediate and free-of-charge access to their health data in electronic form, presented in a comprehensible and commonly used format. Access avenues range from patient portals to computers and smartphones, with compliance with the European Accessibility Act ensuring accessibility for individuals with disabilities. Additionally, individuals have the right to share their electronic health data with other healthcare professionals when transitioning between hospitals. They can also contribute data to their electronic health records, either for personal use or on behalf of trusted individuals like their children. Online platforms facilitate the correction of erroneous data, while the right to restrict access to one’s health data is granted, with exceptions in cases of vital interest. Furthermore, individuals are entitled to easily obtain information regarding the professionals who accessed their data. Member States play a crucial role by designating digital health authorities tasked with enforcing these rights.

Moreover, the EHDS closely aligns with the GDPR in defining how health data can lawfully be processed for secondary use (Hendolin 2022). Specifically, it refers to GDPR Articles 6 and 9 for guidance. The legal foundation for secondary use is outlined in GDPR Article 9(2)(g)–(j). According to the EHDS framework, data users must demonstrate compliance with GDPR Article 6(1)(e) or (f). This means they must prove that accessing health data is necessary to perform a task carried out in the public interest or a legitimate interest. In this regulatory context, data holders, who process data following GDPR Article 6(1)(c), must disclose this information to health data access bodies. These access bodies play a crucial role in ensuring that access to health data is granted based on the specific grounds outlined in the access application, thereby adhering to the principles of lawful data processing (Marcus et al. 2022).

The EHDS proposal upholds the principle of purpose limitation, as stipulated in GDPR Article 5(1b). This principle dictates that personal data must be collected for “specified, explicit, and legitimate purposes” and should not be further processed in a manner that is incompatible with these purposes. In the context of secondary use, data controllers are responsible for evaluating whether further processing is compatible with the original purpose. They must consider the factors outlined in GDPR Article 6(4) unless the new purpose meets certain conditions, such as (1) it is necessary for the performance of a task carried out in the public interest; (2) it is subject to archival purposes in the public interest, scientific research, or historical purposes, as defined in GDPR Article 89(1); and (3) it has the explicit consent of the data subject. These conditions provide a clear framework for ensuring that the secondary use of health data respects the principles of data protection and maintains a strong foundation in legal and ethical standards, while also serving the broader interests of society, research, and innovation.

Even though the EHDS has an ambitious goal, it also confronts a multifaceted array of challenges as it endeavors to harmonize and optimize the use of health data across the diverse landscape of European Member States. One of the most prominent hurdles arises from the inherent disparities in the development of digital health and health data gathering capabilities across different nations within the EU (Molnár-Gábor et al. 2022). These differences stem from variations in administrative systems, cultural norms, and policy-making approaches (EIT Health 2023). The result is a complex landscape where progress in the realm of healthcare data is far from uniform. In addition, at the heart of the challenges lies the intricate web of European legislation and its implementation at the national level. Navigating these complexities requires a delicate balance of tailoring the EHDS implementation to fit the diverse contexts of individual Member States while maintaining the overarching principles of European law. The EHDS illustrates an interoperable framework for data governance. Interoperability and security are set to be mandatory requirements, requiring manufacturers of electronic health record systems to certify their compliance with these standards. Building the necessary infrastructure for the EHDS is a daunting task, especially given the unique intricacies of the healthcare sector. Achieving interoperability, ensuring secure data access, and safeguarding data privacy are all critical technical aspects that must be meticulously addressed to ensure the EHDS operates seamlessly (Shabani and Yilmaz 2022).

Furthermore, engagement with and participation of stakeholders are integral aspects of the EHDS, given the complexity of the healthcare ecosystem involving individuals, healthcare providers, researchers, policymakers, and more. The primary challenge lies in cultivating active involvement and considering the diverse perspectives of these stakeholders. Establishing connections and fostering cooperation are imperative for the EHDS to build the necessary trust and garner support for its success. The EHDS signifies a paradigm shift in health data management, challenging conventional concepts of data governance. The second challenge centers around defining and safeguarding individual rights over their data, recognizing the legitimate interests of various stakeholders, and outlining the responsibilities of data handlers. Striking a delicate balance between data access and protection is crucial. Addressing these challenges goes beyond technical and legal considerations; it is a societal imperative. Confronting these issues directly not only establishes responsible data governance but also has the potential to enhance public support and provide clarity to all stakeholders. This, in turn, increases the likelihood of the EHDS fulfilling its intended purpose.

## A Comparative Perspective

Taiwan and the European Union exhibit both commonalities and differences in their approaches to data governance in the healthcare sector. One of the crucial distinctions between Taiwan and the EU lies in their legal frameworks. Taiwan’s data governance initiatives are primarily governed by its national legal framework and regulations, including its own set of data protection laws. In contrast, the EHDS operates within the broader legal context of the EU, notably under the comprehensive GDPR (Kohl 2022). This legal distinction underscores the complex nature of the EHDS as it navigates the multifaceted legal landscape within the EU (Chronaki 2021). While Taiwan’s data governance framework operates under a single national legal umbrella, the EHDS must harmonize data governance practices across diverse EU member states, each with its own distinct healthcare systems and regulatory frameworks. This necessitates a meticulous alignment of data practices with the GDPR’s stringent requirements, adding an additional layer of complexity to the EHDS initiative as it seeks to maintain legal compliance across a diverse and dynamic European legal environment (Terzis and Santamaria Echeverria 2023).

In addition, cultural norms, administrative systems, and policy-making approaches also contribute to the differentiation between Taiwan and the EHDS. These divergences, intrinsic to each region, have the potential to influence how data governance is implemented and perceived. The EHDS must navigate the diversity of contexts among EU member states, whereas Taiwan’s data governance strategies can be more directly tailored to its unique cultural and administrative landscape. Moreover, the variation in healthcare systems between Taiwan and EU member states adds another layer of distinction. Differences in healthcare structure, funding mechanisms, and service delivery models can impact the generation, access, and utilization of health data. As the EHDS works to harmonize data governance across these varying systems, Taiwan’s approach can be more aligned with its specific healthcare system. Technical infrastructure for data access and sharing is yet another factor of differentiation. While the EHDS aims to establish a unified infrastructure for data access, the existing systems within individual EU countries may vary (Shabani 2022). Taiwan’s data governance framework may exhibit greater centralization and uniformity in this regard.

Despite these differences, Taiwan and the EHDS also share some commonalities in their data governance approaches, which could potentially serve as building blocks for global data governance standards. Both initiatives emphasize the importance of individual autonomy over health data, recognizing that individuals should have greater control and agency over how their personal health information is accessed and used. This alignment reflects a growing global trend toward prioritizing individual privacy and data rights, a foundational principle that underpins modern data governance frameworks (Pagallo 2022).

Another shared aspect between Taiwan and the EHDS is the emphasis on legislative efforts to establish legal clarity, particularly concerning the secondary use of health data, especially when commercial entities are involved. Both jurisdictions recognize the need for robust legal frameworks to regulate and govern the use of health data for various purposes, including innovation. By clearly defining the rules and boundaries for data usage, they aim to strike a balance between fostering innovation and protecting individual privacy and data security.

Furthermore, both Taiwan and the EHDS introduce data governance procedures and organizations to ensure the effective and responsible use of health data. These governance structures act as safeguards to monitor and enforce compliance with data protection and privacy regulations. They play a critical role in overseeing data access, usage, and security, thereby contributing to the overall integrity and trustworthiness of health data ecosystems (Hendolin 2022). However, it is crucial to recognize that the introduction of more bureaucracy could also impede efficiency in data access, ultimately posing a threat to innovation. Adopting a proactive approach is essential to foster a resilient innovation ecosystem, benefiting not just individual innovators but also contributing to the overall economic and technological advancement of the region.

The experiences of Taiwan and the EU indicate a possible alignment in their trajectories. Taiwan’s model emerges from a court challenge, while the EHDS develops through collaborative efforts at the EU level. Despite their unique beginnings, both approaches are gradually being implemented across the diverse contexts of Taiwan and the EU member states. The shared element is the incremental advancement toward secondary use frameworks, shaped by different catalysts and unfolding at varying speeds across jurisdictions.

Last but not least, both Taiwan and the EHDS shall underscore the pivotal role of trust in effective data governance. Despite the ambitious objectives and potential benefits of the EHDS, its success fundamentally depends on building trust among diverse stakeholders, encompassing individuals, healthcare providers, and regulatory bodies. Trust stands as a cornerstone for ensuring broad participation, facilitating data sharing, and fostering mutual recognition of permits across health data access bodies in different Member States. In the absence of a solid foundation of trust, apprehensions related to privacy, security, and the ethical use of health data may pose challenges to the EHDS’s objectives. Thus, the cultivation and enduring maintenance of trust emerge as critical determinants in the secondary use of health data in Taiwan and in ensuring the success and acceptance of the EHDS as a collaborative platform for health data exchange within the European Union.

In summary, Taiwan and EHDS demonstrate alignment in their approach to data governance, particularly in prioritizing individual data autonomy, relying on legislative efforts for legal clarity, and introducing robust data governance procedures. These shared principles and practices could indeed serve as a foundation for the development of global data governance standards, as countries and regions worldwide grapple with the challenges and opportunities presented by the evolving data landscape. However, while legal clarity is a fundamental aspect of establishing frameworks for data governance, it does not necessarily equate to efforts aimed at building public trust. Even with comprehensive laws in place, socio-ethical challenges persist that extend beyond the realm of legal regulations. Gaining public trust requires proactive measures that go beyond mere compliance with the law. Socio-ethical challenges include addressing concerns related to data privacy, ensuring transparency, and actively engaging with the public to foster understanding. The potential misuse of health data, the unequal distribution of benefits, and the ethical implications of emerging technologies are among the complex issues that demand attention. Therefore, alongside legal frameworks, a concerted effort to navigate and mitigate these socio-ethical challenges is essential to establish a foundation of trust and ensure the responsible and ethical implementation of health data systems such as Taiwan and the EHDS.

In the context of Taiwan’s experiences, where the constitutional implications add an extra layer of complexity, gaining public trust becomes paramount. The success of health databases relies not just on compliance with legal frameworks but on individuals being willing to contribute their data voluntarily. Given Taiwan’s constitutional challenge in the secondary use of data, it becomes even more crucial to address socio-ethical concerns and foster an environment where individuals are not only aware of the legal protections but also trust the system enough to remain in the database, actively contributing to the collective health data pool rather than opting out. This underscores the importance of bridging the gap between legal provisions and public perception to ensure the sustained effectiveness and ethical use of health data systems.

Good governance, in this context, extends beyond adhering to laws and regulations to encompass fostering an environment of openness, actively involving stakeholders, and addressing societal concerns. In the specific cases of Taiwan and Europe, good governance needs to be exemplified by clear communication, meaningful engagement with the public, and the establishment of mechanisms for oversight and accountability. Transparent processes for decision-making and the responsible handling of health data contribute to building trust. Additionally, ensuring equitable access to benefits derived from health data and safeguarding privacy are crucial aspects of good governance.

In Taiwan, good governance might also involve a responsive approach to community concerns, regular updates on data usage, and mechanisms for public input. In Europe, where diverse member states are involved, good governance may include harmonizing practices across borders, ensuring representation from various stakeholders, and facilitating collaboration among different health information systems. Ultimately, trustworthiness is a dynamic concept that requires continuous efforts to align actions with ethical standards, engage stakeholders, and address the evolving expectations of the public. It goes beyond legal compliance to build a foundation of credibility and confidence in the responsible management of health data.

## Conclusion

In the rapidly evolving landscape of medical AI development, the secondary use of health data emerges as a critical cornerstone for innovation and progress. This examination of data governance in the contexts of Taiwan and the EU underscores a pivotal lesson: the importance of robust and transparent data governance frameworks, particularly when commercial entities rely on large-scale health data. Commercial entities play an increasingly prominent role in the advancement of medical AI, and their access to vast health data repositories carries great potential for groundbreaking discoveries. However, this access should be underpinned by a trustworthy and transparent data governance framework. Such a framework not only respects individuals’ autonomy over their health data but also ensures that individuals comprehend how their data is accessed and utilized by these entities.

Crucially, this trust and transparency are central to garnering individuals’ willingness to share their data for medical AI development. When individuals are confident that their data is handled responsibly, ethically, and in alignment with their rights and preferences, they become more inclined to contribute to this valuable resource. In turn, this collective willingness to share data forms the bedrock of a trustworthy data ecosystem. The cross-regional examination of Taiwan and the EU highlights that data governance is not just a regulatory necessity; it is a pivotal enabler of progress and innovation in the realm of medical AI. It demonstrates that while technological advancements are essential, an equally profound transformation must occur in the way we respect, protect, and harness health data.
