# Correction to: Secondary Use of Health Data for Medical AI: A Cross‑Regional Examination of Taiwan and the EU

**DOI:** 10.1007/s41649-024-00318-0

**Published:** 2024-08-16

**Authors:** Chih‑hsing Ho

**Affiliations:** Institute of European and American Studies, Academia Sinica, Taipei, Taiwan

**Correction to: Asian Bioethics Review (2024) 16(3):407–422**

10.1007/s41649-024-00279-4

The article “Secondary Use of Health Data for Medical AI: A Cross‑Regional Examination of Taiwan and the EU”, written by Chih‑hsing Ho, was originally published Online First without Open Access. After publication in volume 16, issue 3, pages 407–422, the author decided to opt for Open Choice and to make the article an Open Access publication. Therefore, the copyright of the article has been changed to © The Author(s) 2024, and the article is forthwith distributed under the terms of the Creative Commons Attribution 4.0 International License, which permits the use, sharing, adaptation, distribution and reproduction in any medium or format, as long as you give appropriate credit to the original author(s) and the source, provide a link to the Creative Commons licence, and indicate if changes were made. The images or other third party material in this article are included in the article’s Creative Commons licence, unless indicated otherwise in a credit line to the material. If material is not included in the article’s Creative Commons licence and your intended use is not permitted by statutory regulation or exceeds the permitted use, you will need to obtain permission directly from the copyright holder. To view a copy of this licence, visit http://creativecommons.org/licenses/by/4.0. Open access funding enabled and organized by Projekt DEAL.

Also, the statement in the Funding information section was updated from ‘This research is partially funded by the National Science and Technology Council (NSTC) of Taiwan with the grant number: 112-2410-H-001-011-MY2.’ to ‘This research is partially funded by the National Science and Technology Council (NSTC) of Taiwan with the grant number: 112-2410-H-001-011-MY2, and the Academia Sinica Grand Challenge Programme Seed Grant Project with the grant number: AS-GCS-113-H04.’.

The original article has been corrected.

